# Supplementary material for: Genetic and ecological drivers of molt in a migratory bird
Source: Sci Rep. 2023 Jan 16;13:814. doi: 10.1038/s41598-022-26973-7 (PMC9842746; doi:10.1038/s41598-022-26973-7)
Supplement: Supplementary file 1 — Supplementary Legends. [file 41598_2022_26973_MOESM1_ESM.docx]

**Supplementary Figures - Captions**

**Figure S1.** Mapping gene–environment correlations of molt-migration phenotype across the Painted Bunting breeding range. **(**A) Top ranked environmental variables underlying molt-migration genotypes. (B) Principal components analysis of gradient forest transformed climate variables. Colors are based upon modelled gene– environment correlations from 100 000 random points across the breeding range. Arrows show the loadings of the top-ranked uncorrelated environmental variables. (C) Gradient forest-transformed climate variables from the PCA mapped to geography support climate adaptation across the breeding range. Black dots designating approximate population locations. (D) Histogram of R^2^ of 1000 gradient forest runs of environmental variable randomizations demonstrates the variables BIO15, BIO01, BIO13 and NDVIstd are significantly correlated to the candidate molt-migration genetic variation (pink line is average R^2^ of real data).

**Figure S2**. Statistical association of A) *CSPG4* allele frequency with BIO15 (Precipitation Seasonality) and B) *GLI2* allele frequency with BIO01 (Annual Mean Temperature) across 13 populations sequenced with RAD-Seq.  There was moderate association of C) *CSGP4* allele frequency and BIO01 (Annual Mean Temp) and D) *ARHGAP26* with NDVIstd (variation in Productivity).

**Supplementary Tables - Captions**

**Table S1**. RAD-Seq sampling locations with Latitude, Longitude, number of individuals sequenced before filtering for missingness (N_RAD_nofilter_), the number of individuals retained after filtering (N_RAD_filter_), and number of individuals for which stable isotope analysis defined molt-migration phenotype (N_Phenotyped_) .

**Table S2**. List of samples used for generating the population violin plots in Fig 2 – panel A.

**Table S3**. List of samples used for generating the cluster violin plots in Fig 2 – panel C.

**Table S4**. Number of Painted Buntings successfully screened at the *GLI2* locus at each location across the species breeding range. Site ID correspond to the map code of breeding populations in Fig. 3.
