# Supplementary figures and images for: Genetic and ecological drivers of molt in a migratory bird
Source: Sci Rep. 2023 Jan 16;13:814. doi: 10.1038/s41598-022-26973-7 (PMC9842746; doi:10.1038/s41598-022-26973-7)

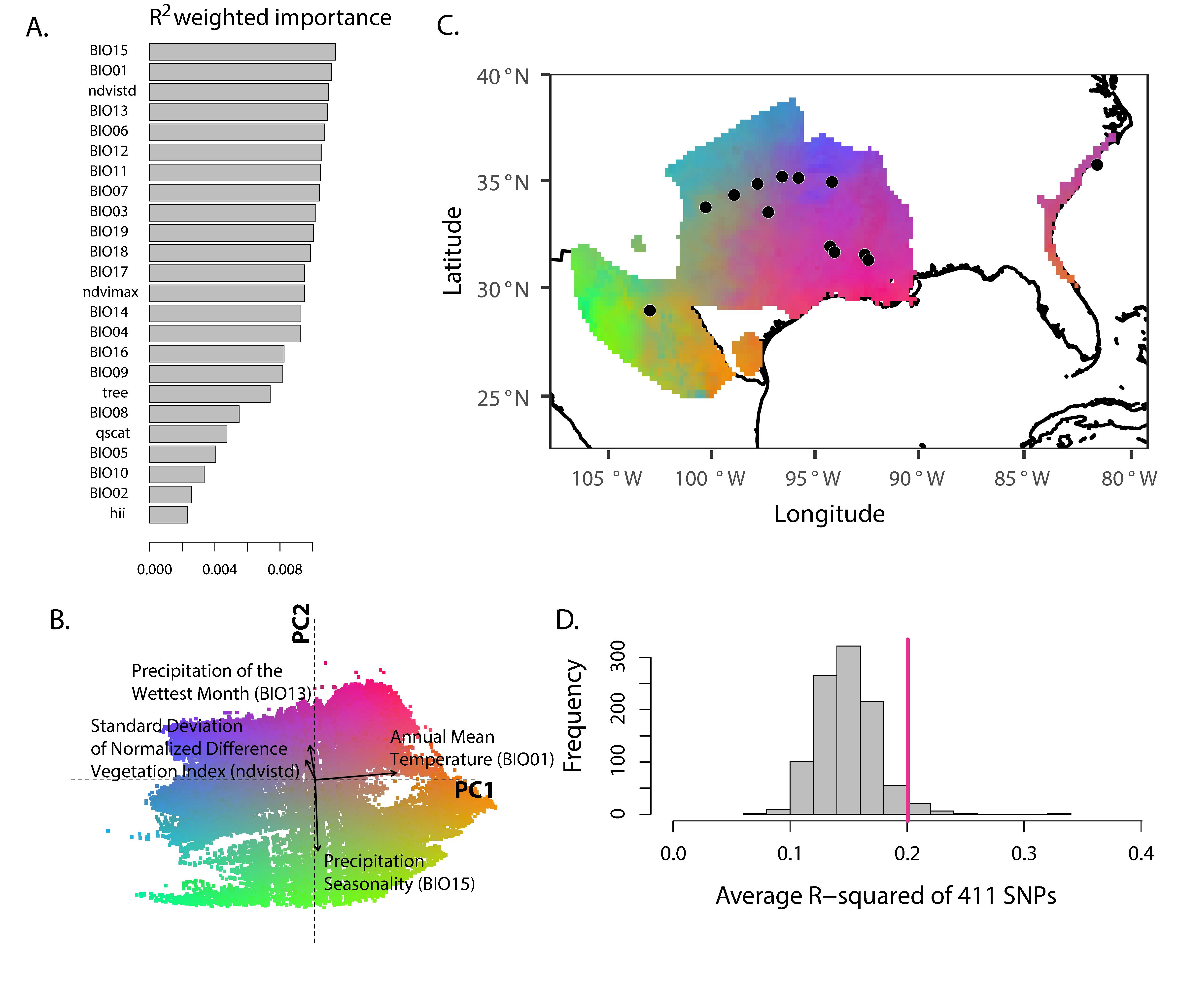

Supplement: Supplementary file 2 — Supplementary Figure S1. [file 41598_2022_26973_MOESM2_ESM.jpeg]

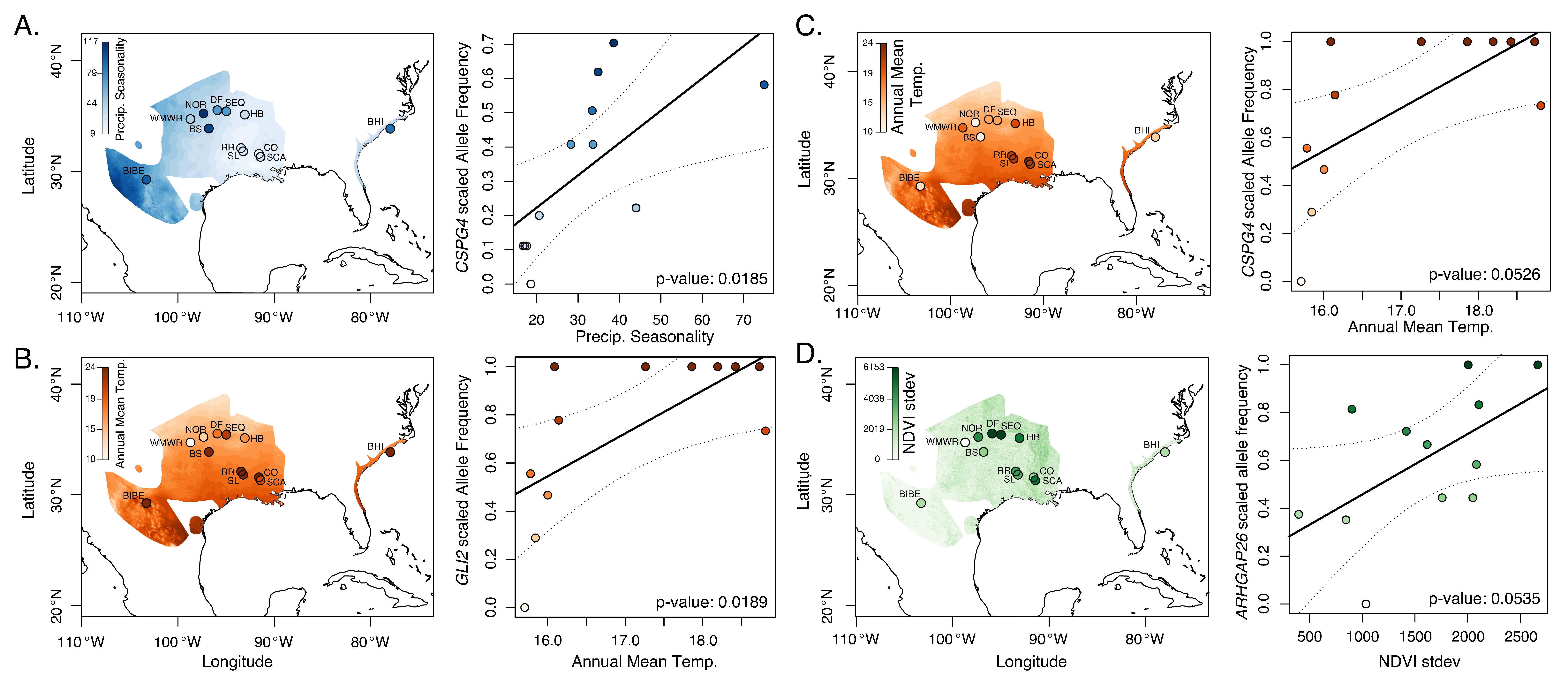

Supplement: Supplementary file 3 — Supplementary Figure S2. [file 41598_2022_26973_MOESM3_ESM.jpeg]
